# *Salmonella enterica* in Northern Italy: Insights from a Historical Collection

**DOI:** 10.3390/pathogens15070771

**Published:** 2026-07-22

**Authors:** Priscilla Pasutto, Antonella Amendola, Maria Gori, Clara Fappani, Marta Gusmeroli, Daniela Colzani, Elisa Borghi, Mirella Pontello, Elisabetta Tanzi, Silvia Bianchi

**Affiliations:** 1 Department of Health Sciences, Università degli Studi di Milano, Via Antonio di Rudinì, 8, 20142 Milan, Italy; priscilla.pasutto@unimi.it (P.P.); antonella.amendola@unimi.it (A.A.); maria.gori@unimi.it (M.G.); clara.fappani@unimi.it (C.F.); marta.gusmeroli@studenti.unimi.it (M.G.); daniela.colzani@unimi.it (D.C.); elisa.borghi@unimi.it (E.B.); mirella.pontello@gmail.com (M.P.); elisabetta.tanzi@unimi.it (E.T.); 2 Coordinated Research Center “EpiSoMI”, Università degli Studi di Milano, Via Antonio di Rudinì, 8, 20142 Milan, Italy

**Keywords:** historical collection, non-typhoidal *Salmonella*, invasiveness, antimicrobial resistance

## Abstract

Historical microbiological collections linked to epidemiological and clinical metadata represent a valuable resource for retrospective investigation of long-term changes in pathogen populations. In this retrospective study, we analyzed the epidemiological, clinical, and microbiological information recorded in the datasheets accompanying *Salmonella enterica* isolates archived in a historical collection assembled in Lombardy, Italy, between 2001 and 2016. Overall, metadata associated with 6624 salmonellosis cases with confirmed serovar identification were included to describe serovar distribution, bloodstream infections, and antimicrobial resistance (AMR) patterns within the collection. Four serovars (*S.* Typhimurium, *S.* Enteritidis, *S.* 1,4,[5],12:i:-, *S.* Napoli) accounted for approximately 80% of all cases. The relative representation of *S.* Napoli increased over the study period, whereas *S.* Choleraesuis showed the highest proportion of bloodstream isolates and predominantly affected adults. Overall, resistance to penicillins (46.9%) and tetracyclines (48.0%) was common, whereas resistance to fluoroquinolones (2.9%) and third-generation cephalosporins (<2%) remained low. Among invasive non-typhoidal *Salmonella* (iNTS) infections, 69.3% of isolates were resistant to at least one antimicrobial class, and 34.7% exhibited multidrug resistance (MDR), with fluoroquinolone resistance increasing to 13.9%. These findings highlight the value of historical microbiological collections and their associated metadata for documenting long-term changes in serovar distribution, invasive infections, and AMR, while emphasizing their importance as a resource for future molecular epidemiology and One Health surveillance.

## 1. Introduction

The emergence and re-emergence of infectious pathogens have highlighted the need for a better understanding of microbial evolution and adaptation. In this context, historical collections of microbial isolates, including microbiological biobanks, represent valuable resources for molecular epidemiology, evolutionary studies, and investigations of pathogenicity and antimicrobial resistance (AMR) mechanisms [1,2,3]. These collections offer a unique opportunity to compare contemporary isolates with those collected in the past, enabling retrospective investigations that support both scientific knowledge and public health strategies [3,4]. In addition, they can strengthen surveillance systems by enabling retrospective evaluation of data collection practices, including laboratory workflows, standardized case-report forms, and antimicrobial susceptibility testing (AST) strategies, thereby improving surveillance and disease characterization over time [5,6,7]. Furthermore, historical collections provide an important resource for evaluating the long-term impact of public health interventions, including food safety regulations and animal vaccination programs, and a basis for evaluating the effectiveness of interventions aimed at reducing pathogen spread and hygiene measures aimed at reducing the spread of foodborne pathogens [8].

According to the 2023 One Health Zoonoses Report, salmonellosis remains one of the most frequently reported zoonotic diseases in the European Union, with more than 77,000 confirmed cases, second only to campylobacteriosis [9]. Salmonellosis is caused by bacteria belonging to the genus *Salmonella* (*S.*), a Gram-negative, facultatively anaerobic bacillus within the family *Enterobacteriaceae* [10]. The genus comprises two species, *S. enterica* and *S. bongori*, and more than 2600 serovars defined on the basis of biochemical, antigenic and genomic characteristics [11,12]. Although some serovars are host-restricted, most have a broad host range and infect both humans and animals [13]. Human infection is primarily acquired through the consumption of contaminated foods, including undercooked meat, eggs, dairy products, and fresh products, or through direct contact with infected animals or contaminated environments. The wide range of animal reservoirs, including poultry, cattle, and wildlife, complicates control measures and facilitates the persistence and spread of the pathogens [13,14].

Clinically, *Salmonella* infections are classified as either typhoidal (TS) or non-typhoidal (NTS). Typhoidal serovars are human-restricted and cause typhoid and paratyphoid fevers, whereas NTS serovars infect a broader range of hosts and typically cause self-limiting gastroenteritis that does not require antibiotic treatment [11]. Nevertheless, approximately 5% of NTS infections progress to bloodstream infection, particularly in young children, older adults, and patients with chronic underlying conditions [15]. Invasive infections may result in severe complications, including meningitis, septic arthritis, and osteomyelitis. Certain NTS serovars, particularly *S.* Choleraesuis, *S.* Dublin, and *S.* Napoli, are more frequently associated with invasive disease and therefore often require prompt antimicrobial therapy [16,17,18].

Globally, *S.* Typhimurium has historically been one of the predominant serovars associated with infections in both humans and pigs, although the distribution of *Salmonella* serovars varies considerably across geographical regions [19,20]. In Europe, NTS accounts for the majority of human infections, whereas typhoidal infections are relatively uncommon and are mainly associated with international travel [9,15,21]. Although several Italian studies have investigated serovar distribution and AMR in human, animal, and food isolates, few have used historical microbial collections linked to epidemiological and clinical metadata to jointly investigate long-term changes in serovar distribution, invasiveness and AMR within a single regional setting [22,23].

Understanding the epidemiology, virulence, and transmission dynamics of *S. enterica* requires not only contemporary surveillance data generated within a One Health framework but also access to historical microbial collections that enable retrospective investigations over extended time periods. These collections provide a unique opportunity to document long-term changes in serovar distribution, AMR, and clinical features while preserving biological material for future molecular and genomic studies.

In the present study, we retrospectively analyzed the epidemiological, clinical and microbiological information recorded in the datasheets accompanying *S. enterica* isolates collected from salmonellosis cases occurring in Lombardy, Italy, between 2001 and 2016. These datasheets included information on patient demographics, clinical characteristics, sample sources, serovar identification, and AMR profiles. Using this historical collection and its associated metadata, we aimed to describe the microbiological, clinical, and demographic features documented in the collection over the 16-year study period, with particular emphasis on serovar distribution, invasiveness, and AMR patterns.

## 2. Materials and Methods

**Historical collection.** The historical collection analyzed in this study is archived at the University of Milan and originated in 1989, continuing to expand until 2016. It was established through a passive laboratory-based reporting system involving collaborating hospitals and diagnostic laboratories participating in the Enter-Net Italia laboratory surveillance network, established in 1997 [24]. This retrospective study was based on the analysis of datasheets accompanying *S. enterica* isolates collected in Lombardy, Italy, between 2001 and 2016. Because isolate submission was voluntary, the completeness and coverage of the collection cannot be estimated, and the number and characteristics of archived isolates may have been influenced by differences in laboratory participation, workload, and diagnostic practices over time. Consequently, the collection should be regarded as a historical repository of *S. enterica* isolates and their associated metadata rather than as a population-based sample representative of all salmonellosis cases occurring in Lombardy.

**Data collection from datasheets**. Each archived isolate was accompanied by a standardized datasheet recording the information available at the time of isolation, including patient demographic characteristics, hospitalization status, clinical specimen, serovar identification and antimicrobial susceptibility results, when available. Only records reporting confirmed *S. enterica* serovar identifications were included in the study. When multiple isolates belonging to the same serovar were recovered from a single clinical case, they were considered duplicate observations, and only one record was retained to avoid overrepresentation of individual cases. When isolates from multiple specimens were available for the same clinical case, the specimen considered to be the most representative of the clinical presentation was retained for analysis. Accordingly, cases with bloodstream isolates were classified as invasive infections irrespective of the concomitant isolation of *Salmonella* from stool specimens, whereas cases with stool isolates only were classified as gastrointestinal infections. Antimicrobial susceptibility testing (AST) was not routinely performed for all patients because antibiotic therapy is generally not recommended for uncomplicated cases of NTS. In addition, participating laboratories applied different AST panels during the study period. To improve comparability across isolates and over time, susceptibility results were grouped and analyzed according to antimicrobial classes rather than individual antimicrobial agents. No additional serotyping or antimicrobial susceptibility testing was performed on the archived isolates for the purposes of this study.

**Serovar identification and isolate preservation**. Throughout the study period, serovar identification of all submitted isolates was routinely performed at the CEPIS (Centro Enterobatteri Patogeni per l’Italia Settentrionale) laboratory, at the University of Milan. Serovars were identified according to the White–Kauffmann–Le Minor scheme [25], the internationally recognized reference method for *Salmonella* serovar classification. Isolates were then preserved by stab inoculation in laboratory-prepared soft agar medium and maintained at room temperature as part of the historical microbiological collection archived at the University of Milan. No additional serotyping or microbiological characterization was performed on the archived isolates for the purposes of this study.

**Invasiveness Index**. To provide a comparative measure of the relative frequency of bloodstream infection among *Salmonella* serotypes within the historical collection, an Invasiveness Index (II), as previously described [26], was calculated for each serotype as the ratio between the number of bloodstream infections caused by that serotype and the total number of infections attributed to the same serotype. In this study, bloodstream infection was used as a proxy for invasive salmonellosis.

**Statistical analysis.** Categorical variables were compared using contingency table analysis. Two-by-two contingency tables were analyzed using OpenEpi (version 3.01; https://www.openepi.com, accessed on 5 March 2026). Odds ratios (ORs) with 95% confidence intervals (95% CIs) were calculated to estimate the strength of association. Two-tailed *p*-values were calculated using Fisher’s exact test or the chi-square test with Yates’ continuity correction, as appropriate. A two-sided *p* value < 0.05 was considered statistically significant. For comparisons involving more than two categories, chi-square tests of independence were performed using an r × c contingency table. Categories were combined when necessary to satisfy the assumptions of the chi-square test.

## 3. Results

**Historical Collection Overview**. The historical collection comprised 7064 serotyped *S. enterica* isolates collected between 2001 and 2016, corresponding to 6624 individual salmonellosis cases. The higher number of isolates reflects the preservation of multiple isolates from the same patient. Stool was the most common specimen source (83.5%; 5897/7064), followed by blood (3.6%; 253/7064), urine (0.8%; 54/7064) and other specimen types (0.5%; 35/7064; e.g., pus, bile). Overall, the specimen source was unknown for 825 isolates (11.7%; 825/7064).

**Figure 1:**
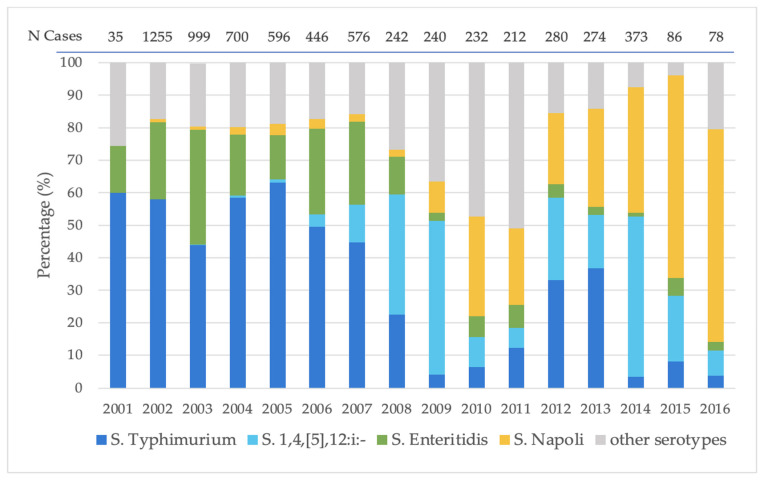
Annual distribution of the main *S. enterica* serovars among serotyped isolates included in the historical collection (2001–2016). Bars show the percentage of each serovar relative to the total number of serotyped isolates available for each year. Numbers above the bars indicate the annual number of serotyped isolates (N). The distribution of serovars varied over time: *S.* Typhimurium predominated in the early years of the study period, while *S.* Enteritidis was consistently represented until 2007 and then declined, whereas the proportions of the monophasic variant *S.* 1,4,[5],12:i:- and *S.* Napoli increased in later years. Annual differences should be interpreted considering the variable number of isolates available for each year.

**Serotype Distribution.** Among the 6624 salmonellosis cases included in the study, 6566 (99%; 6566/6624) were caused by NTS belonging to 164 different serotypes, whereas 58 cases were caused by typhoidal serotypes (*S.* Typhi, *S.* Paratyphi A, B and C) (Supplementary Table S1). *S.* Typhimurium was the most frequently identified serotype (41.8%; 2772/6624), followed by *S.* Enteritidis (18.5%; 1223/6624), *S.* 1,4,[5],12:i:- (9.8%; 649/6624), and *S.* Napoli (9.6%; 636/6624). Overall, these four serotypes were identified in 79.7% (5280/6624) of all salmonellosis cases included in the study. Beyond these four predominant serotypes, *S.* Derby, *S.* Infantis, *S.* Choleraesuis, *S.* Muenchen, *S.* Goldcoast, and *S.* Virchow were collectively associated with a further 511 cases (7.7%). The relative distribution of the four most frequent serotypes changed over the study period (Figure 1). *S.* Typhimurium predominated during the early years, whereas the relative proportions of its monophasic variant (*S.* 1,4,[5],12:i:-) increased in the later years. Conversely, the relative contribution of *S.* Enteritidis declined over time, while *S.* Napoli showed an increasing relative representation after 2009.

**Figure 2:**
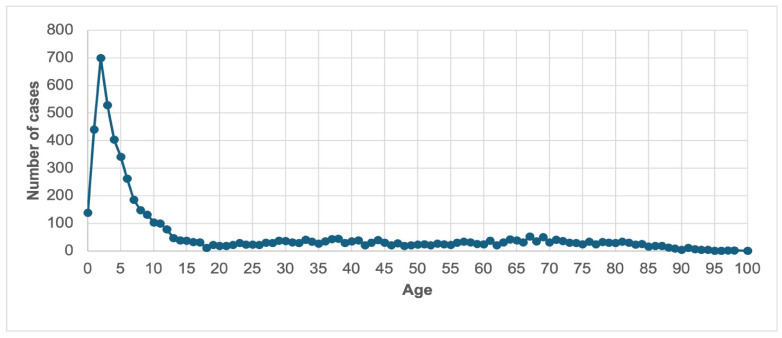
Age distribution of clinical cases associated with *S. enterica* isolates included in the historical collection (Lombardy, 2001–2016). A peak is present at 2 years of age with 700 cases.

**Demographic and clinical characteristics.** Because data completeness varied across the archived datasheets, each analysis was carried out using the available data for the variable of interest. Among cases with available demographic information, 51.8% (3265/6304) occurred in male patients. The age distribution showed a marked peak at 2 years of age (Figure 2), and children 0–4 years accounted for the largest proportion of cases (37.7%; 2213/5870; *p* value < 0.0001; 0–4 age group vs. all other groups). Several serotypes showed distinct age-associated distributions (Table 1). *S.* Typhimurium was more frequently identified among children aged 0–4 years and 5–14 years (OR 1.78, 95% CI 1.59–1.99, *p* < 0.001; OR 1.21, 95% CI 1.07–1.36, *p* = 0.002, respectively). Conversely, *S.* Choleraesuis, *S.* Derby, *S.* Goldcoast and *S.* Muenchen were more frequently identified among patients older than 65 years (OR 16.09, 95% CI 9.80–26.40, *p* < 0.001; OR 2.85, 95% CI 1.95–4.15, *p* < 0.001; OR 3.53, 95% CI 1.95–6.38, *p* < 0.001; OR 2.63, 95% CI 1.58–4.39, *p* < 0.001, respectively) (Table 1 and Supplementary Table S2). Among patients aged 15–39 and 40–64 years, *S.* Enteritidis was more frequently identified than the other NTS serotypes (OR 2.80, 95% CI 2.37–3.30, *p* < 0.001; OR 1.80, 95% CI 1.51–2.15, *p* < 0.001, respectively).

**Travel and food exposure.** Travel history was available for 1361 of the 6624 salmonellosis cases (20.5%), of which 194 (14.3%) were associated with travel. The travel destination was reported for 142 cases, including domestic travel within Italy (62.7%; 89/142) and international travel (37.3%; 53/142). *S.* Typhimurium and *S.* Enteritidis were the serotypes most frequently identified among travel-associated cases. Information on food exposure was available for 466 cases (7.0%; 466/6624), while a specific suspected food item was reported for 300 cases (Table 2). Eggs and egg-containing foods were the most frequently reported exposure, followed by meat, fish and seafood products. Because no microbiological confirmation of *Salmonella* isolation from suspected food items was available, these data should be interpreted as patient-reported exposures only.

**Clinical presentation.** Information allowing classification of the clinical presentation was available for 5853 cases. Based on criteria described in the Section 2, 5529 cases (94.5%; 5529/5853) were classified as gastrointestinal infections, whereas 244 (4.2%; 244/5853) were classified as bloodstream infections. Among bloodstream isolates, *S.* Choleraesuis (24.6%; 60/244), *S.* Typhimurium (22.5%; 55/244) and *S.* Napoli (15.6%; 38/244) were the most frequently identified serotypes (Table 1).

**Invasiveness.** The highest Invasiveness Index (II) was observed for *S.* Choleraesuis (76.9%; 60/78), followed by *S.* Napoli (8.1%; 38/470) and *S.* Typhimurium (2.2%; 55/2518). *S.* Typhi accounted for 22 of the 244 bloodstream isolates (9.0%) and showed an II of 64.7% (22/34). A significant association was observed between serotype and the proportion of bloodstream isolates (*p* < 0.0001).

**Hospitalization.** Hospitalization data were available for 4345 cases, of which 1821 (41.9%) required hospital care. Hospitalization rates among the four most common serotypes ranged from 35.5% to 45.8%. (Table 1). *S.* Choleraesuis showed the highest hospitalization rate compared to all NTS (92.1%, 58/63 vs. 41.9%, 1821/4345; OR 16.07, 95% CI 6.89–45.4, *p* < 0.0001), comparable to that observed for *S.* Typhi (87.5%, 21/24 vs. 41.9%, 1821/4345, OR 9.70, 95% CI 3.17–40.9, *p* < 0.0001).

**Association between age and source of isolation.** A significant association was observed between age group and source of isolation (*p* < 0.001). Children aged 0–4 years were more likely to have stool isolates (42.0%, 656/1562, OR 5.64, 95% CI 3.48–9.14, *p* < 0.001), whereas patients older than 65 years were more likely to have bloodstream isolates (49.1%, 82/167, OR 4.60, 95% CI 3.28–6.46, *p* < 0.001).

**Antimicrobial resistance.** Antimicrobial susceptibility results were available for 5057 cases (76.3%). Because participating laboratories used different antimicrobial susceptibility testing panels during the study period, resistance was analyzed by antimicrobial class. Consequently, the number of isolates tested varied across antimicrobial classes (Table 3 and Supplementary Figure S1). High resistance rates were recorded for penicillins (46.9%; 2000/4264), tetracyclines (48%; 651/1355), and aminoglycosides (28.3%; 927/3272), whereas resistance to third-generation cephalosporins (1.5%; 41/2680) and carbapenems (0.7%; 13/1768) remained low. AMR analyses were further restricted to invasive non-typhoidal *Salmonella* (iNTS) infections. AMR profiles were available for 179 of the 213 (84.0%) iNTS cases (Supplementary Figure S2). Among these, 124 (69.3%, 124/179) were resistant to at least one antibiotic class, whereas 55 (30.7%, 55/179) were susceptible to all tested antimicrobial classes. Among the 124 antimicrobial-resistant iNTS isolates, *S.* Choleraesuis was the most frequently identified serotype (35.5%; 44/124), followed by *S.* Typhimurium (31.5%; 39/124) and *S.* Napoli (11.3%; 14/124). Of the resistant isolates, 39 (31.5%) were resistant to one antibiotic class, 42 (33.9%) to two classes, and 43 (34.7%) to three or more classes. The highest resistance rate was observed for penicillins (93.3%; 98/105), followed by aminoglycosides (73.1%; 49/67), sulfonamides (46%; 40/87) and tetracyclines (46.4%; 26/56), whereas no resistance was detected to carbapenems (0/31) or third-generation cephalosporins (0/81). Resistance to fluoroquinolones was observed in 13.9% (15/108) of iNTS isolates, markedly higher than observed among all isolates with available AMR profiles (2.9%, 136/4711; OR 5.43, 95% CI 2.97–9.42, *p* < 0.0001). Fluoroquinolone resistance was further characterized by serotype distribution. Among the 136 fluoroquinolone-resistant isolates, ciprofloxacin was the most frequently tested antimicrobial (112/136; 82.4%). Of these, 42 isolates (37.5%) belonged to *S.* Napoli, 12 (10.7%) to *S.* Typhimurium, and 11 (9.8%) to *S.* 1,4,[5],12:i:-.

## 4. Discussion

This study presents a retrospective analysis of a large historical collection of *S. enterica* in Italy, comprising strains collected from 6624 cases occurring in Lombardy between 2001 and 2016. By integrating microbiological data with epidemiological and clinical information recorded in the original datasheets, this study provides a retrospective overview of serovar distribution, bloodstream infections, and antimicrobial resistance over a 16-year period. The findings should be interpreted in the context of the study design, as the collection was assembled through passive laboratory-based surveillance and therefore was not intended to represent all salmonellosis cases occurring in Lombardy. Globally, *S.* Typhimurium and *S.* Enteritidis are the two serotypes most frequently associated with human salmonellosis worldwide [14,27]. In the present study, the distributions of three of the four predominant serotypes (*S.* Enteritidis, *S.* Typhimurium, and its monophasic variant *S.* 1,4,[5],12:i:-) were consistent with those reported by the Italian and European surveillance systems during the study period [28,29,30]. However, unlike in most European countries, where *S.* Enteritidis remained the predominant serotype, surveillance data from the Enter-Net Italy network showed that *S.* Typhimurium and its monophasic variant became the most frequently isolated serotypes in human infections after 2008 [31,32]. The serotype distribution observed in this collection was consistent with these national surveillance data [33].

Within the collection, *S.* Typhimurium and *S.* Enteritidis were the most frequently represented serotypes during the early 2000s. After 2007, however, the relative proportions of isolates belonging to these serotypes declined, whereas S. 1,4,[5],12:i:- and S. Napoli became progressively more represented. The apparent decline in S. Typhimurium may partly reflect the separate reporting of its monophasic variant, S. 1,4,[5],12:i:-, included in European surveillance systems from 2010 onwards [34]. Likewise, the reduced proportion of *S*. Enteritidis observed in our collection is consistent with the marked decline in human *S.* Enteritidis infections reported in Italy and across Europe following the implementation of coordinated European and national *Salmonella* control programs in poultry and laying hens [35,36].

The emergence of *S.* Napoli within the collection deserves particular attention. Across Europe, from 2000 to 2013, the incidence of *S.* Napoli increased by 256%, making it the 13th most frequently isolated serovar from human cases [37,38,39]. In the present collection, *S.* Napoli was the most frequently identified serotype among iNTS isolates collected during the period 2010–2014, accounting for 15.8% of invasive cases. Genomic and phylogenetic analyses indicated that *S.* Napoli belongs to the *S.* Typhi subclade of clade A, showing a close relationship with *S.* Paratyphi A and sharing genes associated with the typical typhoid fever virulence [18]. Together, these observations support the epidemiological and clinical relevance of this serotype. The most recent data indicate that *S.* Napoli remains an emerging serotype in Europe, with most reported infections concentrated in a limited number of countries, particularly Italy, France and Switzerland [40]. Within this European context, Italy represents one of the countries most affected by this serotype [41]. These findings, together with the association between *S.* Napoli and ciprofloxacin resistance observed in our collection, highlight the potential public health importance of this serotype and support the need for its continued surveillance in Italy.

Among non-typhoidal serovars, *S.* Choleraesuis showed the highest II (76.9%) and hospitalization rate (92.1%). Unlike the other predominant serotypes, which were mainly associated with gastrointestinal infections in young children, *S.* Choleraesuis was predominantly identified in bloodstream isolates from older adults [42,43]. This observation is consistent with previous findings that highlighted it not only as a swine-associated serotype but as a significant cause of invasive salmonellosis in humans [16,44]. Its close evolutionary relationship with *S.* Paratyphi C may help explain its enhanced invasiveness [45,46,47]. From a public health perspective, these findings highlight the importance of maintaining surveillance systems for *S. enterica* and ensuring systematic monitoring of invasive serotypes.

The antimicrobial resistance profile observed in the collection reflected the resistance patterns circulating during the study period. High levels of resistance to penicillins and tetracyclines (>45%) limited the therapeutic value of these antimicrobial classes and were consistent with the persistence of resistance to these antibiotics in Italy. According to the most recent ECDC and EFSA report, approximately 40% of *Salmonella* isolates in Italy remain resistant to these antimicrobial classes, whereas lower proportions are reported in many other European countries [48]. In contrast, resistance to fluoroquinolones and sulfonamides (2.9% and 10.2%, respectively) was lower than that currently reported in Italy (9.1% and 43.7%, respectively) and Europe (21.8% and 20.8%, respectively), suggesting that resistance to these antimicrobial classes has increased over time [33,48]. Notably, fluoroquinolone resistance reached 13.9% among iNTS isolates in our collection, emphasizing the clinical relevance of invasive *Salmonella* infections [49].

In particular, the increase in fluoroquinolone resistance is of particular concern, as fluoroquinolone-resistant non-typhoidal *Salmonella* has recently been included in the WHO list of high-priority bacterial pathogens [50]. In Italy, aminoglycosides were the only antimicrobial classes showing a lower resistance rate in the historical collection than that currently reported (28.3% vs. 5.2%), whereas resistance to third- and fourth-generation cephalosporins and carbapenems has remained low, supporting the continued effectiveness of these antibiotic classes. Importantly, one-third of iNTS isolates were multidrug-resistant, underscoring the importance of continuous AMR surveillance to support empirical therapy and antimicrobial stewardship.

This study has several limitations. The collection was generated through a passive laboratory-based reporting system and therefore does not represent a population-based sample of salmonellosis cases in Lombardy. In addition, the availability of demographic and clinical information varied over time. The incomplete availability of epidemiological and clinical information, particularly for travel history, food exposure, and hospitalization, may have influenced the strength of some subgroup analyses and should be considered when interpreting the findings. AST was performed by multiple participating laboratories, and although results were interpreted according to the standards in use at the time, differences in testing methodologies and changes in interpretative criteria over the study period may have affected the comparability of AMR data. Although grouping susceptibility results according to antimicrobial classes improved comparability across laboratories and years, some methodological heterogeneity cannot be excluded. Consequently, the findings should be interpreted as descriptive of the archived collection rather than representative of the epidemiology of salmonellosis in the region.

Despite these limitations, historical microbiological collections linked to epidemiological and microbiological metadata represent a valuable resource for retrospective investigations of serotype distribution and antimicrobial resistance. Recent whole-genome sequencing studies have considerably advanced the understanding of *Salmonella* evolution, virulence, and antimicrobial resistance [51,52]. Historical collections such as the one described here provide an important complementary resource by preserving well-documented archived isolates and associated metadata that can support future genomic investigations within a One Health framework.

## 5. Conclusions

This study highlights the value of a historical microbiological collection and its associated metadata for retrospectively investigating long-term changes in *S. enterica* serotype distribution, bloodstream infections, and antimicrobial resistance. The findings identify *S.* Napoli and *S.* Choleraesuis as serotypes of public health relevance and support the importance of continued surveillance of invasive *Salmonella* infections and AMR. More broadly, this study reinforces the scientific value of preserving historical microbiological collections as resources for future molecular epidemiology and One Health surveillance.

## Figures and Tables

**Table 1:** Demographic, clinical and microbiological characteristics of *S. enterica* cases, stratified by the six most relevant serotypes of the study. Data are presented as number (percentage). Percentages were calculated using the number of cases with available data for each variable.

	All Serotypes	*S.* Typhimurium	*S.* Enteritidis	*S.* 1,4,[5],12:i:-	*S*. Napoli	*S.* Choleraesuis	*S*. Typhi
N Cases (%)	6624 (100)	2772 (41.8)	1223 (18.5)	649 (9.80)	636 (9.60)	82 (1.24)	37 (0.6)
**Sex: N (%)**							
Available data	6304	2693	1199	531	570	81	32
M	3265 (51.8)	1432 (53.1)	596 (49.7)	280 (52.7)	300 (52.6)	51(63.0)	25 (78.1)
**Age: N (%)**							
Available data	5870	2566	1146	420	499	76	29
0–4 age	2213 (37.7)	1163 (45.3)	301 (26.3)	183 (43.6)	205 (41.1)	0 (0)	4 (13.8)
5–14 age	1438 (24.5)	679 (26.5)	283 (24.7)	119 (28.3)	159 (31.9)	4 (5.3)	7 (24.1)
15–39 age	736 (12.5)	242 (9.4)	269 (23.5)	38 (9.0)	24 (4.8)	3 (3.9)	13 (44,8)
40–64 age	704 (12.0)	233 (9.1)	202 (17.6)	31 (7.4)	38 (7.6)	16 (21.1)	5 (17.2)
>65 age	779 (13.3)	249 (9.7)	91 (7.9)	49 (11.7)	73 (14.6)	53 (69.7)	0 (0.0)
**Source of isolation: N (%)**							
Available data	5853	2518	1150	433	470	78	34
Stool	5529 (94.5)	2443 (97.0)	1119 (97.3)	426 (98.4)	430 (91.4)	8(10.3)	12 (35.3)
Blood	244 (4.2)	55 (2.2)	25 (2.2)	5 (1.2)	38 (8.1)	60 (76.9)	22 (64.7)
Others *	80(1.4)	20 (0.8)	6 (0.5)	2 (0.5)	2 (0.4)	10 (12.8)	0 (0.0)
**Hospitalization: N (%)**							
Available data	4345	1919	889	253	353	63	24
Hospitalized	1821 (41.9)	796 (41.5)	316 (35.5)	116 (45.8)	157 (44.5)	58 (92.1)	21 (87.5)

Note: * Specimens included urine, bile, abscess material, pus.

**Table 2:** Food and water sources involved in *S. enterica* infections.

Implicated Food and Water (%)	
Available data	300
Water	3 (1.0)
Meat	58 (19.3)
Crustaceans and mollusks	21 (7.0)
Fish	18 (6.0)
Milk, butter and derivatives	12 (4.0)
Eggs	67 (22.3)
Long-life canned foods	2 (0.7)
Fresh vegetables	2 (0.7)
Composite Food—eggs and milk	101 (33.7)
Composite Food—eggs	16 (5.3)

**Table 3:** Antibiotic resistance of *S. enterica* strains obtained from the datasheets. Resistance percentages were calculated using the number of isolates tested for each antimicrobial class as the denominator. The number of isolates tested varied according to the antimicrobial susceptibility panels routinely adopted during the study period (2001–2016).

Antibiotic Classes	Antimicrobials Included	Isolates Tested (N)	Resistant Isolates, N (%)
**Beta-lactams**			
Penicillins	Amoxicillin, Ampicillin, Cloxacillin, Oxacillin, Mezlocillin, Piperacillin, Ticarcillin.	4264	2000 (46.9)
Penicillins + β-lactamase inhibitor	Ampicillin/Sulbactam, Piperacillin/Tazobactam, Ticarcillin/Clavulanic acid	1729	383 (22.2)
First-generation cephalosporins	Cephalothin, Cefazolin	3329	565 (17.0)
Second-generation cephalosporins	Cefuroxime, Cefuroxime Axetil, Cefoxitin	936	251(26.8)
Third-generation cephalosporins	Cefotaxime, Ceftazidime, Ceftriaxone, Cefixime, Cefpodoxime, Ceftiroxime	2680	41 (1.5)
Fourth-generation cephalosporins	Only Cefepime tested	925	20 (2.2)
Monobactams	Only Aztreonam tested	788	11(1.4)
Carbapenems	Imipenem, Meropenem, Ertapenem	1768	13 (0.7)
**Fluoroquinolones/Quinolones**	Ciprofloxacin, Levofloxacin, Norfloxacin, Ofloxacin	4711	136 (2.9)
**Aminoglycosides**	Amikacin, Gentamicin, Netylminicin, Tobramycin, Streptomycin, Kanamycin	3272	927 (28.3)
**Tetracyclines/Glycylcyclines**	Tetracycline, Doxycycline, Tigecycline	1355	651(48.0)
**Sulfonamides**	Cotrimoxazole, Trimethoprim, Sulfamethoxazole, Trimethoprim/Sulfamethoxazole	4722	480 (10.2)
**Phenicols**	Only Chloramphenicol	668	112 (16.8)
**Polymyxins**	Only Colistin tested	98	0 (0.0)
**Macrolides**	Only Erythromycin tested	4	2 (50.0)
**Others**	Fosfomycin	150	4 (2.7)
	Nitrofurantoin	27	4 (14.8)

## Data Availability

Data supporting the findings of this study are not publicly available due to institutional restrictions and the presence of sensitive epidemiological information but are available from the corresponding author upon reasonable request.

## Supplementary Materials

The following supporting information can be downloaded at https://www.mdpi.com/article/10.3390/pathogens15070771/s1. Figure S1: Percentage of antibiotic resistance among *S. enterica* isolates collected from 2001 to 2016. Figure S2: Percentage of AMR profiles in iNTS. Table S1: Number of cases and percentage of 168 serotypes recorded in the period 2001–2016. Table S2: Distribution of minor serotypes (*S.* Derby, *S.* Goldcoast and *S.* Muenchen) per age.

## Abbreviations

The following abbreviations are used in this manuscript:

AST	Antimicrobial susceptibility testing
AMR	Antimicrobial resistance
BPPL	Bacterial Priority Pathogens list
CI	Confidence intervals
ECDC	European Centre for Disease Prevention and Control
EFSA	European Food Safety Authority
EU	European Union
II	Invasiveness Index
iNTS	Invasive non-typhoidal *Salmonella*
MDR	Multiple-drug resistance
NTS	Non-typhoidal *Salmonella*
ORs	Odds ratios
TS	Typhoidal *Salmonella*
WGS	Whole-genome sequencing

## References

[1] De Paoli P. (2005). Bio-banking in microbiology: From sample collection to epidemiology, diagnosis and research. FEMS Microbiol. Rev..

[2] Müller H., Dagher G., Loibner M., Stumptner C., Kungl P., Zatloukal K. (2020). Biobanks for life sciences and personalized medicine: Importance of standardization, biosafety, biosecurity, and data management. Curr. Opin. Biotechnol..

[3] Medina P.B., Kealy J., Kozlakidis Z. (2022). Integrating research infrastructures into infectious diseases surveillance operations: Focus on biobanks. Biosaf. Health.

[4] Muzambi R., Bhaskaran K., Rentsch C.T., Smeeth L., Brayne C., Garfield V., Williams D.M., Chaturvedi N., Warren-Gash C. (2022). Are infections associated with cognitive decline and neuroimaging outcomes? A historical cohort study using data from the UK Biobank study linked to electronic health records. Transl. Psychiatry.

[5] Delpy L., Astbury C.C., Aenishaenslin C., Ruckert A., Penney T.L., Wiktorowicz M., Ciss M., Benko R., Bordier M. (2024). Integrated surveillance systems for antibiotic resistance in a One Health context: A scoping review. BMC Public Health.

[6] Gentile B., Grottola A., Orlando G., Fregni Serpini G., Venturelli C., Meschiari M., Anselmo A., Fillo S., Fortunato A., Lista F. (2020). A retrospective whole-genome sequencing analysis of carbapenem and colistin-resistant Klebsiella pneumoniae nosocomial strains isolated during an MDR surveillance program. Antibiotics.

[7] Postiglione U., Batisti Biffignandi G., Corbella M., Merla C., Olivieri E., Petazzoni G., Feil E.J., Bandi C., Cambieri P., Gaiarsa S. (2023). Combining genome surveillance and metadata to characterize the diversity of Staphylococcus aureus circulating in an Italian hospital over a 9-year period. Microbiol. Spectr..

[8] Rodrigues da Costa M., Pessoa J., Meemken D., Nesbakken T. (2021). A systematic review on the effectiveness of pre-harvest meat safety interventions in pig herds to control Salmonella and other foodborne pathogens. Microorganisms.

[9] European Food Safety Authority (EFSA), European Centre for Disease Prevention and Control (ECDC) (2023). The European Union One Health 2022 Zoonoses Report. EFSA J..

[10] Han J., Aljahdali N., Zhao S., Tang H., Harbottle H., Hoffmann M., Frye J.G., Foley S.L. (2024). Infection biology of Salmonella enterica. EcoSal Plus.

[11] Brenner F.W., Villar R.G., Angulo F.J., Tauxe R., Swaminathan B. (2000). Salmonella nomenclature. J. Clin. Microbiol..

[12] Chattaway M.A., Langridge G.C., Wain J. (2021). Salmonella nomenclature in the genomic era: A time for change. Sci. Rep..

[13] Galán-Relaño Á., Valero Díaz A., Huerta Lorenzo B., Gómez-Gascón L., Mena Rodríguez M.Á., Carrasco Jiménez E., Pérez Rodríguez F., Astorga Márquez R.J. (2023). Salmonella and salmonellosis: An update on public health implications and control strategies. Animals.

[14] World Health Organization (WHO) Salmonella. https://www.who.int/health-topics/salmonella.

[15] European Centre for Disease Prevention and Control (ECDC) Salmonellosis. Annual Epidemiological Report for 2022. https://www.ecdc.europa.eu/en/publications-data/salmonellosis-annual-epidemiological-report-2022.

[16] Chiu C.-H., Su L.-H., Chu C. (2004). Salmonella enterica serotype Choleraesuis: Epidemiology, pathogenesis, clinical disease, and treatment. Clin. Microbiol. Rev..

[17] Mangat C.S., Bekal S., Avery B.P., Côté G., Daignault D., Doualla-Bell F., Finley R., Lefebvre B., Bharat A., Parmley E.J. (2019). Genomic investigation of the emergence of invasive multidrug-resistant Salmonella enterica serovar Dublin in humans and animals in Canada. Antimicrob. Agents Chemother..

[18] Huedo P., Gori M., Zolin A., Amato E., Ciceri G., Bossi A., Pontello M. (2017). Salmonella enterica serotype Napoli is the first cause of invasive nontyphoidal salmonellosis in Lombardy, Italy (2010–2014), and belongs to Typhi subclade. Foodborne Pathog. Dis..

[19] Wang Y., Liu Y., Lyu N., Li Z., Ma S., Cao D., Pan Y., Hu Y., Huang H., Gao G.F. (2023). The temporal dynamics of antimicrobial-resistant Salmonella enterica and predominant serovars in China. Natl. Sci. Rev..

[20] Ferrari R.G., Rosario D.K.A., Cunha-Neto A., Mano S.B., Figueiredo E.E.S., Conte-Junior C.A. (2019). Worldwide epidemiology of Salmonella serovars in animal-based foods: A meta-analysis. Appl. Environ. Microbiol..

[21] Westrell T., Monnet D.L., Gossner C., Heuer O., Takkinen J. (2014). Drug-resistant Salmonella enterica serotype Kentucky in Europe. Lancet Infect. Dis..

[22] Pitti M., Garcia-Vozmediano A., Tramuta C., Maurella C., Decastelli L., CeRTiS Clinical Laboratories Group (2023). Monitoring of antimicrobial resistance of Salmonella serotypes isolated from humans in Northwest Italy, 2012–2021. Pathogens.

[23] Petrin S., Orsini M., Massaro A., Olsen J.E., Barco L., Losasso C. (2023). Phenotypic and genotypic antimicrobial resistance correlation and plasmid characterization in Salmonella spp. isolates from Italy reveal high heterogeneity among serovars. Front. Public Health.

[24] Gorietti S., Busani L., on behalf of the Gruppo ENTER-NET Italia (2001). Salmonella Surveillance in 2000: The Italian ENTER-NET System. Bollettino Epidemiologico Nazionale. https://www.epicentro.iss.it/ben/2001/novembre/2_en.

[25] Issenhuth-Jeanjean S., Roggentin P., Mikoleit M., Guibourdenche M., de Pinna E., Nair S., Fields P.I., Weill F.-X. (2014). Supplement 2008–2010 (No. 48) to the White–Kauffmann–Le Minor scheme. Res. Microbiol..

[26] Parisi A., Crump J.A., Stafford R., Glass K., Howden B.P., Kirk M.D. (2019). Increasing incidence of invasive nontyphoidal Salmonella infections in Queensland, Australia, 2007–2016. PLoS Negl. Trop. Dis..

[27] Feasey N.A., Dougan G., Kingsley R.A., Heyderman R.S., Gordon M.A. (2012). Invasive non-typhoidal Salmonella disease: An emerging and neglected tropical disease in Africa. Lancet.

[28] European Food Safety Authority (EFSA) (2009). The Community Summary Report on Trends and Sources of Zoonoses and Zoonotic Agents in the European Union in 2007. EFSA J..

[29] European Centre for Disease Prevention and Control (ECDC) (2010). Annual Epidemiological Report on Communicable Diseases in Europe. https://data.europa.eu/doi/10.2900/35039.

[30] European Centre for Disease Prevention and Control (ECDC) Salmonellosis. Annual Epidemiological Report for 2015. https://www.ecdc.europa.eu/en/publications-data/salmonellosis-annual-epidemiological-report-2015.

[31] Dionisi A.M., Filetici E., Owczarek S., Arena S., Benedetti I., Lucarelli C., Luzzi I., Scavia G., Minelli F., Ciaravino G. (2011). ENTER-NET: Sorveglianza delle infezioni trasmesse da alimenti e acqua. Rapporto dell’attività 2007–2009. Not. Ist. Super. Sanità.

[32] García Fernández A., Luzzi I., Dionisi A.M., Owczarek S., Arena S., Lucarelli C., Luzzi I., García Fernández A., Dionisi A.M., Lucarelli C., Gattuso A., Gianfranceschi M.V., Maugliani A., Caprioli A., Morabito S., Scavia G. (2017). Salmonella. Enter-Net Italia e Registro Italiano della Sindrome Emolitico Uremica: Sorveglianza delle Infezioni da Salmonella, Campylobacter, Escherichia coli Produttore di Shiga-Tossina e Listeria monocytogenes (2010–2015).

[33] Lucarelli C., Garcia-Fernandez A., Dionisi A.M., Owczarek S., Arena S., Fortini D., Errico G., Maraglino F., Pilati S., Palamara A.T. Sorveglianza Nazionale Delle Infezioni da Salmonella, Campylobacter, Shigella e Yersinia. Dati Enter-Net Italia 2016–2021. Rapporti ISS Sorveglianza 2024, RIS-1/2024. https://www.iss.it/documents/20126/0/RIS-1-2024.pdf/c2710a8c-fef8-1455-5d6a-90aa978bc4c3?t=1725524713756.

[34] European Centre for Disease Prevention and Control (ECDC) Food- and Waterborne Diseases and Zoonoses. Annual Epidemiological Report 2012. https://www.ecdc.europa.eu/en/publications-data/food-and-waterborne-diseases-and-zoonoses-annual-epidemiological-report-2014-2012.

[35] European Commission Food-Borne Diseases (Zoonoses): Control of Salmonella. https://food.ec.europa.eu/food-safety/biological-safety/food-borne-diseases-zoonoses/control-salmonella_en.

[36] Koutsoumanis K., Allende A., Alvarez-Ordóñez A., Bolton D., Bover-Cid S., Chemaly M., De Cesare A., Herman L., Hilbert F., EFSA Panel on Biological Hazards (BIOHAZ) (2019). Salmonella Control in Poultry Flocks and Its Public Health Impact. EFSA J..

[37] Gori M., Ebranati E., Scaltriti E., Huedo P., Ciceri G., Tanzi E., Pontello M., Zehender G., Pongolini S., Bolzoni L. (2018). High-Resolution Diffusion Pattern of Human Infections by Salmonella enterica Serovar Napoli in Northern Italy Explained through Phylogeography. PLoS ONE.

[38] Graziani C., Luzzi I., Owczarek S., Dionisi A.M., Busani L. (2015). Salmonella enterica Serovar Napoli Infection in Italy from 2000 to 2013: Spatial and Spatio-Temporal Analysis of Case Distribution and the Effect of Human and Animal Density on the Risk of Infection. PLoS ONE.

[39] Huedo P., Gori M., Amato E., Bianchi R., Valerio E., Magnoli L., Pontello M. (2016). A Multischool Outbreak Due to Salmonella enterica Serovar Napoli Associated with Elevated Rates of Hospitalizations and Bacteremia, Milan, Italy, 2014. Foodborne Pathog. Dis..

[40] Sabbatucci M., Dionisi A.M., Pezzotti P., Lucarelli C., Barco L., Mancin M., Luzzi I. (2018). Molecular and Epidemiologic Analysis of Reemergent Salmonella enterica Serovar Napoli, Italy, 2011–2015. Emerg. Infect. Dis..

[41] Leati M., Zaccherini A., Ruocco L., D’Amato S., Busani L., Villa L., Barco L., Ricci A., Cibin V. (2021). The Challenging Task to Select Salmonella Target Serovars in Poultry: The Italian Point of View. Epidemiol. Infect..

[42] Yahav D., Eliakim-Raz N., Leibovici L., Paul M. (2016). Bloodstream Infections in Older Patients. Virulence.

[43] Chen P.-L., Lee H.-C., Lee N.-Y., Wu C.-J., Lin S.-H., Shih H.-I., Lee C.-C., Ko W.-C., Chang C.-M. (2012). Non-Typhoidal Salmonella Bacteraemia in Elderly Patients: An Increased Risk for Endovascular Infections, Osteomyelitis and Mortality. Epidemiol. Infect..

[44] Chiu C.-H., Tang P., Chu C., Hu S., Bao Q., Yu J., Chou Y.-Y., Wang H.-S., Lee Y.-S. (2005). The Genome Sequence of Salmonella enterica Serovar Choleraesuis, a Highly Invasive and Resistant Zoonotic Pathogen. Nucleic Acids Res..

[45] Liu W.-Q., Feng Y., Wang Y., Zou Q.-H., Chen F., Guo J.-T., Peng Y.-H., Jin Y., Li Y.-G., Hu S.-N. (2009). Salmonella Paratyphi C: Genetic Divergence from Salmonella Choleraesuis and Pathogenic Convergence with Salmonella Typhi. PLoS ONE.

[46] Fricke W.F., Mammel M.K., McDermott P.F., Tartera C., White D.G., Leclerc J.E., Ravel J., Cebula T.A. (2011). Comparative Genomics of 28 Salmonella enterica Isolates: Evidence for CRISPR-Mediated Adaptive Sublineage Evolution. J. Bacteriol..

[47] Zhou Z., Lundstrøm I., Tran-Dien A., Duchêne S., Alikhan N.-F., Sergeant M.J., Langridge G., Fotakis A.K., Nair S., Stenøien H.K. (2018). Pan-Genome Analysis of Ancient and Modern Salmonella enterica Demonstrates Genomic Stability of the Invasive Para C Lineage for Millennia. Curr. Biol..

[48] European Food Safety Authority (EFSA), European Centre for Disease Prevention and Control (ECDC) (2025). Plain Language Summary of the European Union Summary Report on Antimicrobial Resistance in Zoonotic and Indicator Bacteria from Humans, Animals and Food in 2022–2023. EFSA J..

[49] Zhan Z., Xie Z., Lin H., Guo J., Cai M., Wu Y., Liang S. (2026). Prevalence of Antibiotic Resistance in Invasive Non-Typhoidal Salmonella across 19 Developing Countries, 2000–2026: A Systematic Review and Meta-Analysis. J. Glob. Antimicrob. Resist..

[50] World Health Organization (WHO) WHO Bacterial Priority Pathogens List, 2024: Bacterial Pathogens of Public Health Importance to Guide Research, Development and Strategies to Prevent and Control Antimicrobial Resistance. https://www.who.int/publications/i/item/9789240093461.

[51] Wang Y., Xu X., Jia S., Qu M., Pei Y., Qiu S., Zhang J., Liu Y., Ma S., Lyu N. (2025). A global atlas and drivers of antimicrobial resistance in Salmonella during 1900–2023. Nat. Commun..

[52] Wang Y., Xu X., Zhu B., Lyu N., Liu Y., Ma S., Jia S., Wan B., Du Y., Zhang G. (2023). Genomic analysis of almost 8000 Salmonella genomes reveals drivers and landscape of antimicrobial resistance in China. Microbiol. Spectr..

