# Supplementary material for: Salmonella enterica in Northern Italy: Insights from a Historical Collection
Source: Pathogens. 2026 Jul 22;15(7):771. doi: 10.3390/pathogens15070771 (PMC13414502; doi:10.3390/pathogens15070771)
Supplement: Supplementary file 1 [file pathogens-15-00771-s001.zip › pathogens-4353225-supplementary3.pdf]

Salmonella enterica in Northern Italy: Insights from a Historical Collection

Priscilla Pasutto[1], *Antonella Amendola*[1,2], *Maria Gori, Clara Fappani, Marta Gusmeroli, Daniela Colzani, Elisa Borghi*[1,2], *Mirella Pontello*[1,2], *Elisabetta Tanzi*[1,2], *Silvia Bianchi*[1,2]*.

[1] Department of Health Sciences, Università degli Studi di Milano
[2] Coordinated Research Center "EpiSoMI", Università degli Studi di Milano
* Correspondence: silvia.bianchi@unimi.it

Figure S1: Percentage of antibiotic resistance of *S. enterica* isolates from 2001-.2016. N represents the total number of isolates tested for antibiotic class.

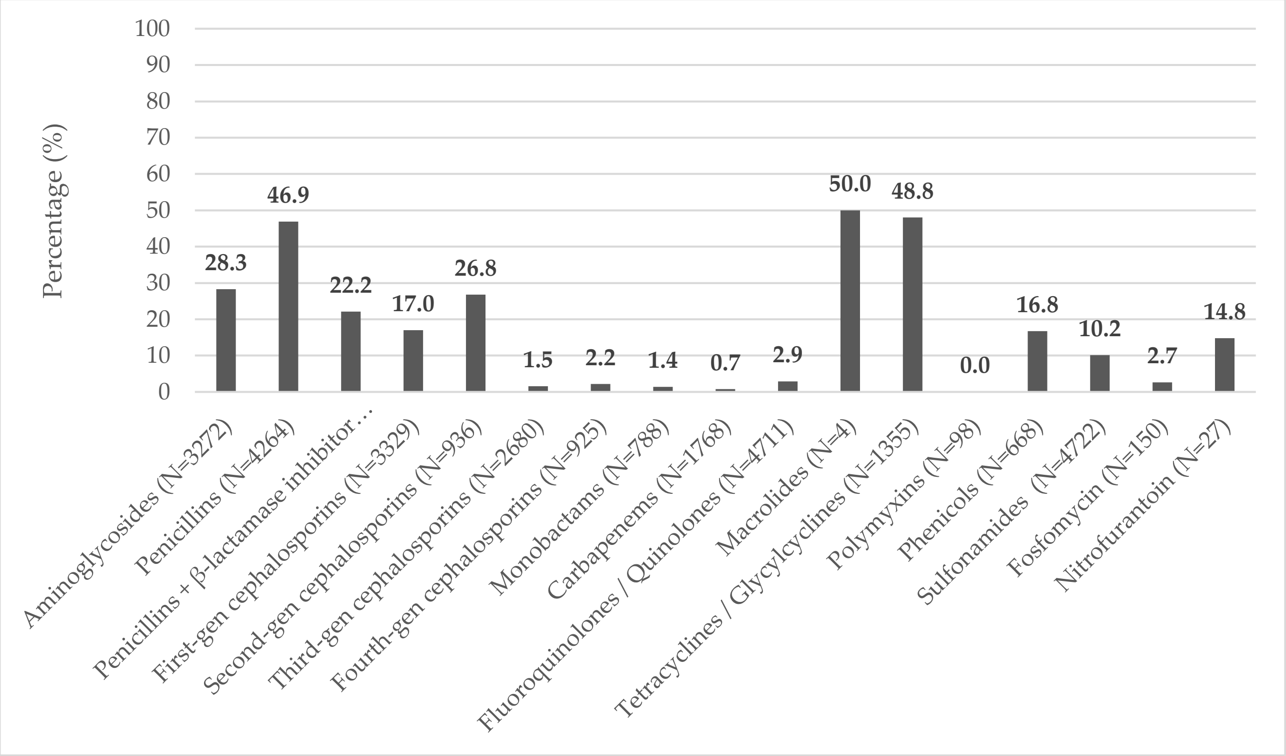

Figure S2: Percentage of AMR profiles in iNTS. N represents the total number of isolates tested for antibiotic class.

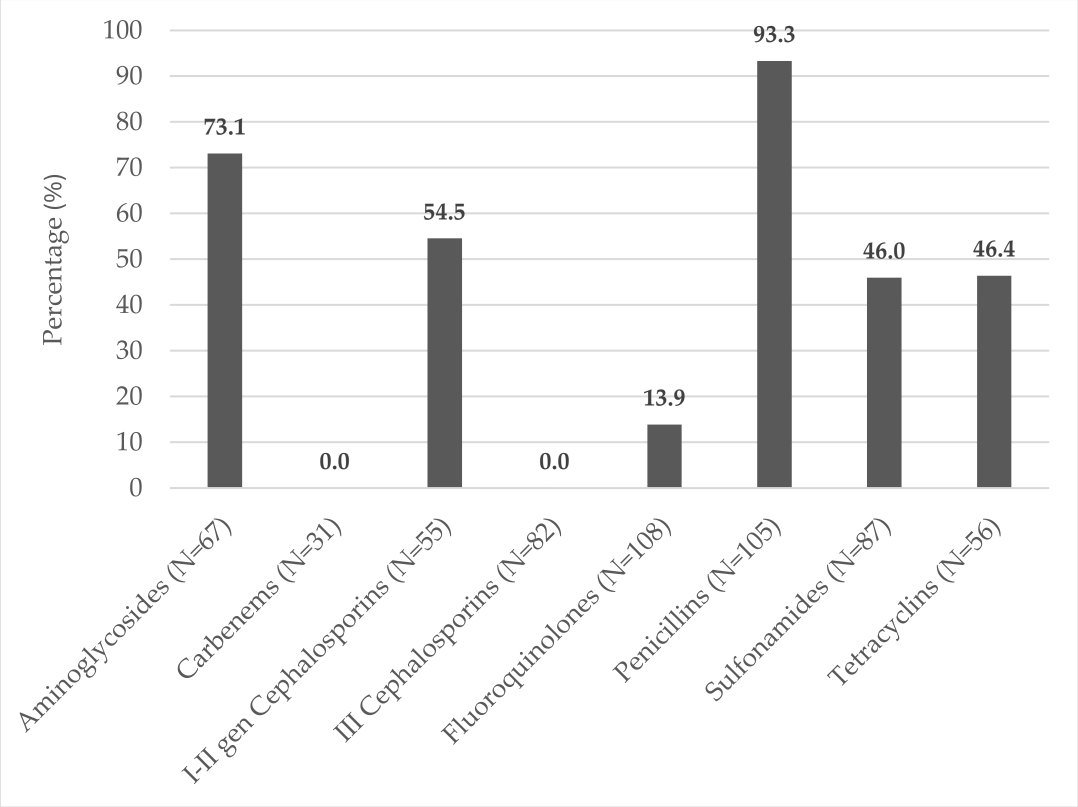

Table S1: Number of cases and percentage of serotypes recorded in the period 2001-2016.

Salmonella enterica serotypes		Number of cases (N)	Percentage (%)
1	Typhimurium	2772	41.8
2	Enteritidis	1223	18.5
3	1,4,[5],12,:i:-	649	9.8
4	Napoli	636	9.6
5	Derby	148	2.2
6	Infantis	86	1.3
7	Choleraesuis	82	1.2
8	Muenchen	76	1.1
9	Goldcoast	60	0.9
10	Virchow	59	0.9
11	Hadar	57	0.9
12	Bovismorbificans	46	0.7
13	Brandenburg	43	0.6
14	London	40	0.6
15	Panama	39	0.6
16	Rissen	39	0.6
17	Typhi	37	0.6
18	Heidelberg	34	0.5
19	Anatum	28	0.4
20	Agona	26	0.4

21	Bredeney	25	0.4
22	Blockley	20	0.3
23	Give	20	0.3
24	Thompson	20	0.3
25	Kottbus	18	0.3
26	Livingstone	18	0.3
27	Saintpaul	15	0.2
28	Stanley	12	0.2
29	Irchel	11	0.2
30	Paratyphi A	10	0.2
31	Zaiman	10	0.2
32	Corvallis	9	0.1
33	Montevideo	9	0.1
34	Paratyphi B	9	0.1
35	Coeln	8	0.1
36	Braenderup	7	0.1
37	Kapemba	7	0.1
38	Arizonae	6	0.1
39	Haifa	6	0.1
40	Kimuenza	6	0.1
41	Manhattan	6	0.1
42	Salamae	6	0.1
43	KentucKy	5	0.1
44	Newport	5	0.1
45	Parkroyal	5	0.1
46	Veneziana	5	0.1
47	Othmarschen	4	0.1
48	Toronto	4	0.1
49	Diarizonae	3	<0.1
50	Indiana	3	<0.1
51	Kedougou	3	<0.1
52	Tsevie	3	<0.1
53	Afula	2	<0.1
54	Agbeni	2	<0.1
55	Akanji	2	<0.1
56	Baildon	2	<0.1
57	Berta	2	<0.1
58	Bispebjerg	2	<0.1
59	Colorado	2	<0.1
60	Fyris	2	<0.1
61	Goettingen	2	<0.1
62	Isangi	2	<0.1
63	Jerusalem	2	<0.1
64	Kasenyi	2	<0.1
65	kitenge	2	<0.1
66	Lagos	2	<0.1

67	Larochelle	2	<0.1
68	Lomalinda	2	<0.1
69	Mathura	2	<0.1
70	Mbandaka	2	<0.1
71	Mendoza	2	<0.1
72	Paratyphi C	2	<0.1
73	Poona	2	<0.1
74	Sangalkam	2	<0.1
75	Senftenberg	2	<0.1
76	Sinstorf	2	<0.1
77	Soerenga	2	<0.1
78	Tallahassee	2	<0.1
79	Uppsala	2	<0.1
80	Warb	2	<0.1
81	Wippra	2	<0.1
82	Zanzibar	2	<0.1
83	Aberdeen	1	<0.1
84	Abony	1	<0.1
85	Admstua	1	<0.1
86	Ahuza	1	<0.1
87	Ajiombo	1	<0.1
88	Albert	1	<0.1
89	Api	1	<0.1
90	Assinie	1	<0.1
91	Ball	1	<0.1
92	Bochum	1	<0.1
93	Bongori	1	<0.1
94	Breukelen	1	<0.1
95	Brikama	1	<0.1
96	Bristock	1	<0.1
97	Bukuru	1	<0.1
98	Calabar	1	<0.1
99	Chailey	1	<0.1
100	Chenna	1	<0.1
101	Chester	1	<0.1
102	Chudleigh	1	<0.1
103	Claibornei	1	<0.1
104	Clerkenwell	1	<0.1
105	Cyprus	1	<0.1
106	Dabou	1	<0.1
107	Dar-es-Salaam	1	<0.1
108	Daula	1	<0.1
109	Eastbourne	1	<0.1
110	EKO	1	<0.1
111	Escanaba	1	<0.1
112	Essen	1	<0.1

113	Farmsen	1	<0.1
114	Galiema	1	<0.1
115	Gallinarum	1	<0.1
116	Glostrup	1	<0.1
117	Goeteborg	1	<0.1
118	Grampian	1	<0.1
119	Herzliya	1	<0.1
120	Hiduddify	1	<0.1
121	Hillingdon	1	<0.1
122	Houston	1	<0.1
123	Inglis	1	<0.1
124	Israel	1	<0.1
125	Kande	1	<0.1
126	Kingston	1	<0.1
127	Kinondoni	1	<0.1
128	Kisangani	1	<0.1
129	Kisii	1	<0.1
130	Kokomlemle	1	<0.1
131	Kuilsriver	1	<0.1
132	Lerum	1	<0.1
133	Litchfield	1	<0.1
134	Lomita	1	<0.1
135	Marmande	1	<0.1
136	Meleagridis	1	<0.1
137	Merston	1	<0.1
138	Muenster	1	<0.1
139	Ndolo	1	<0.1
140	Ohio	1	<0.1
141	Orion	1	<0.1
142	Oritamerin	1	<0.1
143	Pensacola	1	<0.1
144	Pikime	1	<0.1
145	Potsdam	1	<0.1
146	Redougou	1	<0.1
147	Seattle	1	<0.1
148	Shangani	1	<0.1
149	Sofia	1	<0.1
150	Solna	1	<0.1
151	Sterrenbons	1	<0.1
152	Stratchona	1	<0.1
153	Szentes	1	<0.1
154	Telelkebir	1	<0.1
155	Tiko	1	<0.1
156	Umbilo	1	<0.1
157	Urbana	1	<0.1
158	Vellore	1	<0.1

159	Virginia	1	<0.1
160	Welikade	1	<0.1
161	Weltevreden	1	<0.1
162	Wentworth	1	<0.1
163	Westerstede	1	<0.1
164	Westhapton	1	<0.1
165	Worthington	1	<0.1
166	Yoruba	1	<0.1
167	Yundum	1	<0.1
168	Zuilen	1	<0.1
Total		6624	

Table S2: Distribution of minor serotypes (*S.* Derby, *S.* Goldcoast and *S.* Muenchen) per age.

	All serotypes	*S.* Derby	*S.* Muenchen	*S.* Goldcoast
Available data	5870	135	74	49
0-4 age	2213 (37.7)	50 (37.0)	22 (29.7)	10 (20.4)
5-14 age	1438 (24.5)	15 (11.1)	13 (17.6)	8 (16.3)
15-39 age	736 (12.5)	7 (5.2)	8 (10.8)	8 (16.3)
40-64 age	704 (12.0)	23 (17.0)	10 (13.5)	6 (12.2)
>64 age	779 (13.3)	40 (29.6)	21 (28.4)	17 (34.7)
